# Metal complexes of sulfamethazine/benzoin-based Schiff base ligand: synthesis, characterization, DFT calculations, and antimicrobial activities

**DOI:** 10.55730/1300-0152.2729

**Published:** 2024-12-24

**Authors:** Kerem BURAN

**Affiliations:** Department of Pharmaceutical Chemistry, Hamidiye Faculty of Pharmacy, University of Health Sciences, İstanbul, Turkiye

**Keywords:** Sulfamethazine, benzoin, Schiff base, antimicrobial activity, density functional theory calculation

## Abstract

**Background/aim:**

The global rise in bacterial resistance poses a significant challenge, exacerbated by the overuse of antibacterial agents. Schiff base-metal complexes have gained attention for their potential antimicrobial properties, attributed to their unique three-dimensional structures and modes of action, such as cell wall inhibition and membrane disruption. The aim of this study was to synthesize and characterize a novel Schiff base (L) derived from sulfamethazine and benzoin, and to develop metal complexes of it with Cu^2+^, Co^2+^, Ni^2+^, and Cd^2+^. This research sought to evaluate the antibacterial and antifungal activities of the synthesized Schiff base and its metal complexes to explore their potential as effective antimicrobial agents.

**Materials and methods:**

The compound L and its metal complexes were synthesized. The compounds were characterized with ^1^H NMR, ^13^C NMR, mass spectrometry, UV-vis spectroscopy, and FTIR. Antimicrobial activities were tested on *E. coli* ATCC 8739, *S. aureus* ATCC 6538, *P. aeruginosa* ATCC 9027, and *C. albicans* ATCC 10231. To investigate the molecular properties of compound L, density functional theory calculations were also performed.

**Results:**

The synthesized compounds (L, 1, 2, 3, 4) were tested for antibacterial and antifungal activities. Copper-based compound 1 showed the best overall antifungal activity, while zinc-based compound 3 demonstrated notable antibacterial efficacy against *P. aeruginosa*. Although all compounds outperformed L in antifungal tests, none surpassed ciprofloxacin, the reference drug, in antibacterial assays. These results highlight the potential of Schiff base-metal complexes as promising antimicrobial agents.

**Conclusion:**

This study highlights the potential of Schiff base-metal complexes as effective antimicrobial agents in response to the growing challenge of bacterial resistance. The synthesized Schiff base (L) and metal complexes of it with Cu^2+^, Co^2+^, Ni^2+^, and Cd^2+^ exhibited promising antibacterial and antifungal activities, with copper-based compound 1 showing the most potent antifungal action and zinc-based compound 3 demonstrating significant antibacterial efficacy against *P. aeruginosa*. Although none of the compounds outperformed ciprofloxacin in antibacterial assays, all were more effective than the parent Schiff base in antifungal tests. These findings suggest that Schiff base-metal complexes, particularly copper and zinc derivatives, hold considerable promise for developing new antimicrobial agents. Further optimization and testing could enhance their clinical application in combating resistant infections.

## 1. Introduction

**Figure 1:**
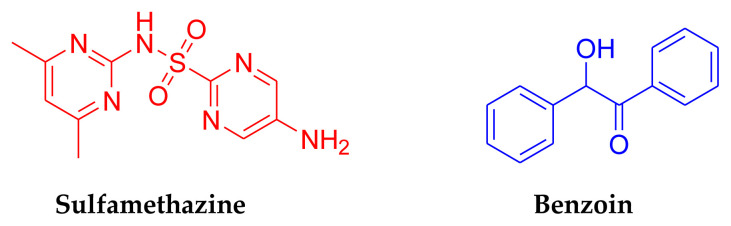
Structures of sulfamethazine and benzoin.

Over the past century, the discovery and development of various antibiotic classes have not been sufficient to combat the ongoing global issue of bacterial resistance (Sabbah et al., 2018; Zafar et al., 2023a). While bacterial resistance is a natural progression, the extensive and often inappropriate use of antibacterial agents in animal farming has artificially accelerated the emergence of resistant strains. This increases the likelihood of bacteria from animals infecting humans (Chang et al., 2015). Historically, bacterial infections have led to high mortality rates worldwide. Fortunately, many natural and synthetic antibiotics have significantly improved human health since the introduction of penicillin as a powerful antibacterial agent in the 1940s. However, treating bacterial infections remains challenging due to the rise of infectious diseases and the increasing number of multidrug-resistant bacterial strains. Despite the availability of numerous antibacterial agents and chemotherapeutics, the ongoing development of resistance among various bacterial strains underscores the urgent need for new classes of antibacterial agents (Verma et al., 2020). In medicinal chemistry, the sulfonamide and sulfonyl functional groups have played a crucial role ever since sulfonamide-containing antibacterial drugs were first discovered (Zafar et al., 2023b). Molecules that contain a sulfonamide group have different biological activities such as anticancer (Akurathi et al., 2014), carbonic anhydrase inhibitory (Supuran et al., 2001), antidiabetic (Buran et al., 2024), and antibacterial activities (Kaya et al., 2013; Ovung and Bhattacharyya, 2021). Sulfamethazine, also known as sulfadimidine, is a sulfonamide antibiotic used primarily in veterinary medicine to treat bacterial infections in animals. It belongs to a class of antibiotics known as sulfa drugs, which work by inhibiting the synthesis of folic acid in bacteria, thereby preventing their growth and multiplication (Kumar Verma et al., 2020). Sulfamethazine (4-amino-*N*-(4,6-dimethylpyrimidin-2-yl)benzenesulfonamide) is effective against a broad range of gram-positive and -negative bacteria (Figure 1) (Ovung and Bhattacharyya, 2021). Another important functional group that is responsible for several biological activities is Schiff bases. Schiff bases composed of an imine group (–C=N) have a broad range of biological activities such as anticancer (Uddin et al., 2020), antimalarial (Meena and Kumar Baroliya, 2023), anti-Alzheimer (Alkahtani et al., 2023), and antimicrobial (Fonkui et al., 2018).

Schiff base-metal complexes are also important and biologically active complexes that are composed of Schiff base and metal atoms. The advantage of Schiff base-metal complexes is that their three-dimensional structures may be more active than small organic molecules. These complexes show a broad range of antimicrobial activities. These activities may be caused by the inhibition of microbial cell wall synthesis and corruption of membrane functions. Ligands play a vital role in the biological activities of metal complexes. Benzoin is a small organic molecule that contains hydroxy and ketone groups, and it is suitable for construction of Schiff base-metal complexes (Figure 1). In the present study, a novel Schiff base (L) was synthesized from sulfamethazine and benzoin. It was characterized by ^1^H NMR, ^13^C NMR, FTIR, UV-vis spectroscopy, and MALDI-TOF-MS. Then metal complexes of L were synthesized with Cu^2+^, Co^2+^, Ni^2+^, and Cd^2+^. The antibacterial and antifungal activities of L and metal complexes were investigated.

## 2. Materials and methods

All the chemicals were purchased from Fluka Chemie AG Buchs and Sigma Aldrich and used without further purification. Thin-layer chromatography (TLC) on silica gel 60 F254 aluminum sheets was used for monitoring reactions. Ethyl acetate and *n*-hexane (1:1) were used as the mobile phase and detection was conducted with UV light. ^1^H NMR and ^13^C NMR spectra were registered in DMSO on an Agilent 400/54 (400 MHz) NMR system. The mass spectra were obtained by MALDI-TOF/MS (Bruker-ultrafleXtreme).

### 2.1. General method for the synthesis of L

Benzoin (1 eq, 2.4 mmol) and sulfamethazine (1 eq, 2.4 mmol)) were mixed in 20 mL of ethanol and refluxed for 6 h. Then the precipitate was filtered and dried.

#### N-(4,6-Dimethyl-pyrimidin-2-yl)-4-(2-hydroxy-1,2-diphenyl-ethylideneamino)-benzenesulfonamide

White solid (71% yield). ^1^H NMR (400 MHz, *DMSO*) δ 11.02 (s, 1H), 7.98 (d, *J* = 7.5 Hz, *2*H), 7.64 (d, *J* = 8.6 Hz, 1H), 7.54 (t, *J* = 7.4 Hz, 1H), 7.42 (dd, *J* = 15.0, 7.5 Hz, *4*H), 7.29 (t, *J* = 7.4 Hz, *2*H), 7.21 (t, *J* = 7.3 Hz, 1H), 6.71 (s, 1H), 6.55 (d, *J* = 8.6 Hz, 1H), 6.05 (dd, *J* = 19.9, 5.8 Hz, *2*H), 5.95 (s, 1H), 2.22 (s, 6H). ^13^C NMR (400 MHz, *DMSO*) δ 199.6, 157.0, 153.3, 140.1, 135.1, 133.67, 130.7, 129.2, 129.0, 128.9, 128.1, 127.7, 112.2, 76.0, 23.5. FT-IR (υ_max_, cm^−1^): 3307–2864 cm^−1^ (OH), 3060 cm^−1^ (C–H_aromatic_), 2922 cm^−1^ (C–H_aliphatic_), 1678 cm^−1^ (C=N–). MALDI-TOF/MS (m/z): Calculated: 472.1569 M^+^, Found: 473.2223 [M+1]^+^.

### 2.2. General method for the synthesis of compounds 1–4

Compound L (2 eq, 0.424 mmol) was reacted with the respective metal salts (1 eq), including CuSO_4_.5H_2_O, CoCl_2_, NiCl_2_.6H_2_O, and Zn(NO_3_)_2_.6H_2_O. Initially, the metal salts (1 eq, 0.212 mmol) were sonicated in an ultrasonic bath for 10 min. Subsequently, the metal salts were mixed with compound L and the mixture was refluxed for 3–5 h. The resulting precipitated solid was obtained by cooling the reaction mixture, followed by filtration and drying.

### 2.3. Antimicrobial activity assay

The compounds were evaluated individually against four microorganisms, including three bacterial species (*E. coli* ATCC 8739, *S. aureus* ATCC 6538, and *P. aeruginosa* ATCC 9027) and one yeast species (*C. albicans* ATCC 10231), all obtained from the American Type Culture Collection (ATCC). The minimum inhibitory concentration (MIC) values were determined using the microdilution method. The bacterial strains were cultured on Mueller–Hinton agar (MHA) and the yeast strain on Sabouraud dextrose agar (SDA). The bacterial cultures were incubated for 24 h at 36 ± 1 °C, while the yeast cultures were incubated for 48 h at 36 ± 1 °C to ensure fresh growth. Each microbial suspension was standardized to match a 0.5 McFarland standard in phosphate-buffered saline (PBS). The compounds were dissolved in methanol to appropriate concentrations and placed into 15-mL sterile test tubes. For the microdilution assay, 96-well plates were prepared by adding 95 μL of broth and 5 μL of inoculum to each well. Mueller–Hinton broth (MHB) was used for the bacterial isolates, and Sabouraud dextrose broth (SDB) for the yeast isolates. From the stock solutions, 100 μL of each compound at the appropriate concentration was initially added to the first wells, followed by serial dilutions across the next five wells. The last well, containing 195 μL of the broth without any compound and 5 μL of the inoculum, served as a negative control. The plates were then incubated at the specified times and temperatures. Microbial growth was measured using an ELx 800 Universal Microplate Reader (Biotek Instruments Inc., Highland Park, VT, USA) by recording absorbance at 600 nm for bacterial isolates and 530 nm for fungal isolates. MIC values were identified as the lowest concentration of each compound that inhibited visible microbial growth.

## 3. Results and discussion

### 3.1. Chemistry

**Figure 2:**
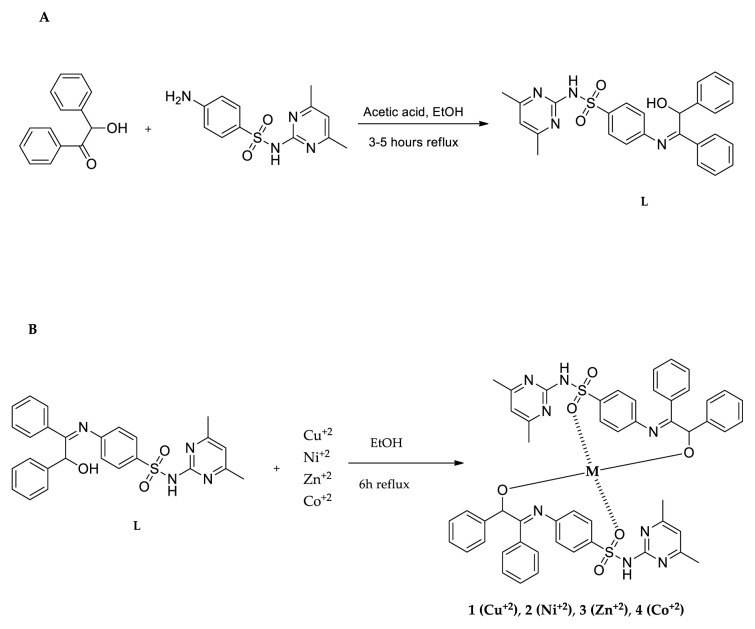
Synthetic pathway of compound L (A) and compounds 1, 2, 3 and 4 (B).

Benzoin and sulfamethazine were used to synthesize a Schiff base (L) (Figure 2). Benzoin (1.0 eq) and sulfamethazine (1.0 eq) were mixed in a glass balloon and the experiment was conducted in ethanol (EtOH) with a catalytic amount of glacial acetic acid for approximately 3–5 h, leading to the synthesis of compound L with a yield of 71%. Subsequently, in the preparation of Schiff base-metal complexes, L (2.0 eq) was reacted with the respective metal salts (1.0 eq) in ethanol under reflux conditions. Both L and its metal complexes underwent characterization using a variety of analytical techniques, including ^1^H NMR, ^13^C NMR, mass spectrometry, UV-vis spectroscopy, and FTIR. The percentage yields of these complexes ranged from 23% to 55%.

### 3.2. Characterization of compounds

**Figure 3:**
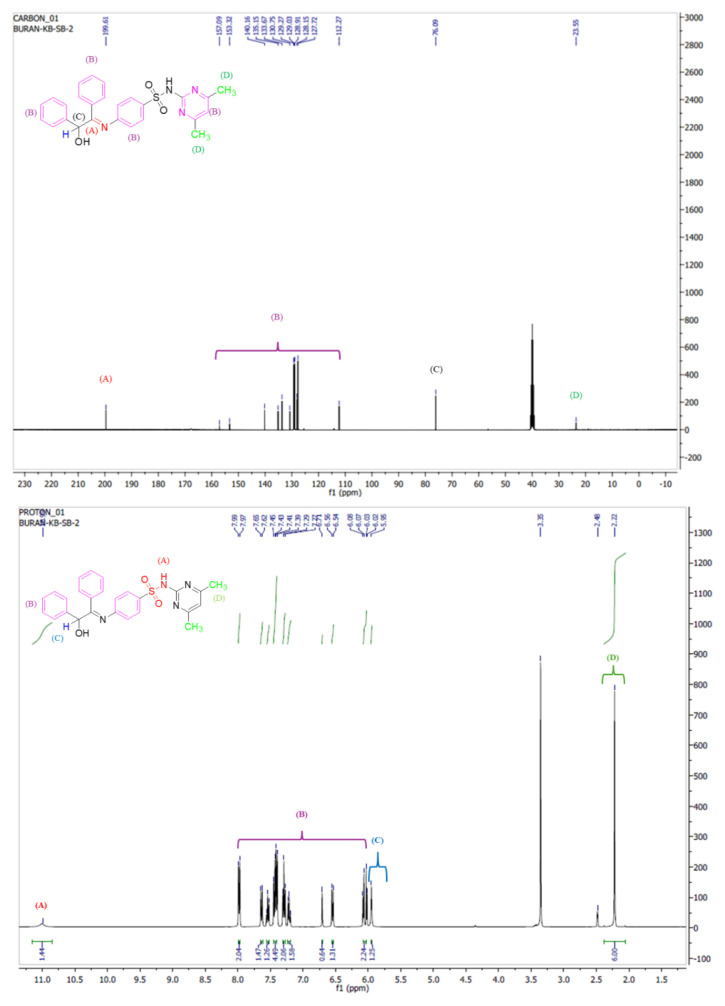
^1^H NMR and ^13^C NMR spectra of compound L.

Compound L was characterized initially using ^1^H NMR and ^13^C NMR spectroscopy. In the ^1^H NMR spectrum, the sulfonamide hydrogen (–SO_2_-N–H) appeared as a singlet at 11.02 ppm. The aromatic protons were observed in the range of 7.98 to 6.05 ppm, while the benzylic proton was detected at 5.95 ppm. The aliphatic –CH_3_ signal was found at 2.22 ppm and the water signal was detected at 3.35 ppm (Figure 3).

**Figure 4:**
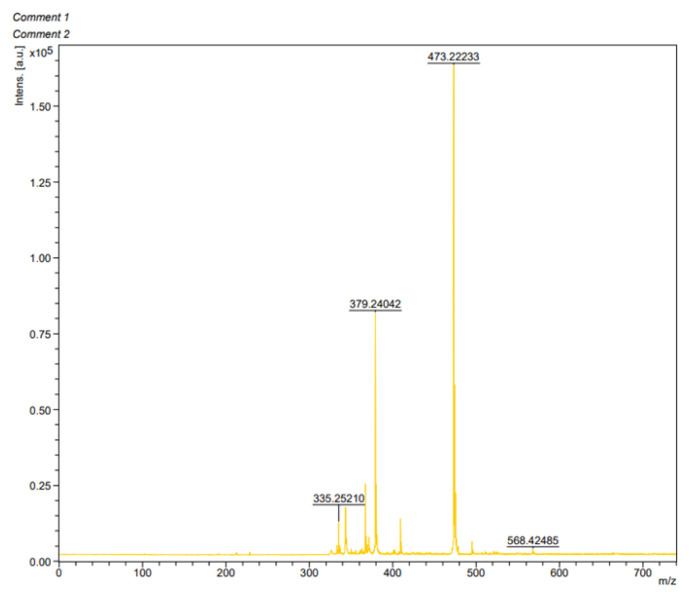
The mass spectrum of L.

The ^13^C NMR spectrum displayed the anticipated peaks for compound L. The carbon atom of the imine group (C=N–) was identified at 199.6 ppm. Aromatic carbons appeared in the range of 157.0–112.2 ppm. The benzylic carbon was detected at 76.0 ppm, while the aliphatic carbon of the methyl groups was observed at 23.5 ppm, as expected (Figure 3). Mass spectrometry analyses were also performed for L (Figure 4). The calculated mass of L M^+^: 472.1569, the MALDI-TOF-MS measurement result was [M+1]^+^: 473.2223.

**Figure 5:**
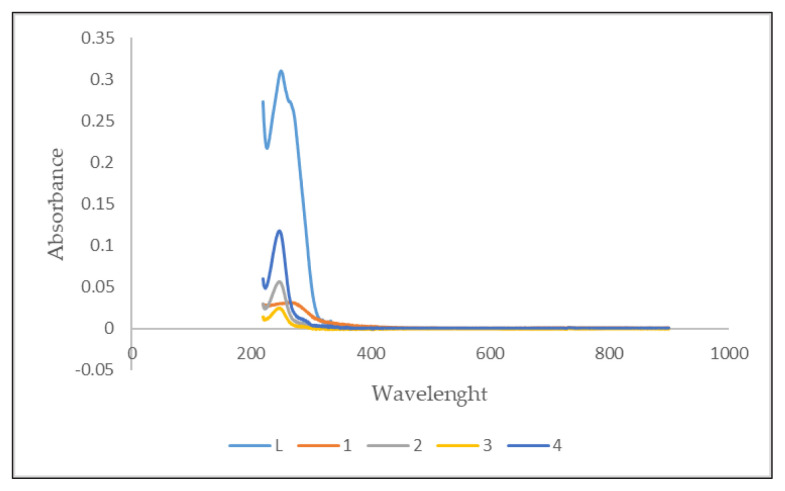
UV spectrum of L, 1, 2, 3, and 4.

In order to investigate the electronic properties of the compounds, UV spectra were analyzed. The examination revealed distinct electronic properties for compounds L, 1, 2, 3, and 4. Ethanol was used as solvent. The UV cut-off value of ethanol is approximately 205 nm; therefore, a wavelength range of 220–900 nm was scanned, revealing that compound L exhibited strong absorption peaks at 254 nm. In contrast, the UV spectra of the Schiff base-metal complexes (1, 2, 3, 4) were shifted a little to the left side. Absorbance peaks were detected at different wavelengths for the compounds studied: 252 nm for compounds 1 and 2, 239 nm for compound 3, and 243 nm for compound 4. The key difference between the absorbance peaks of the ligand (L) and its Schiff base-metal complexes (compounds 1, 2, 3, and 4) was the absorbance intensity. The absorbance intensities of the Schiff base-metal complexes were lower compared to L, as depicted in Figure 5.

**Figure 6:**
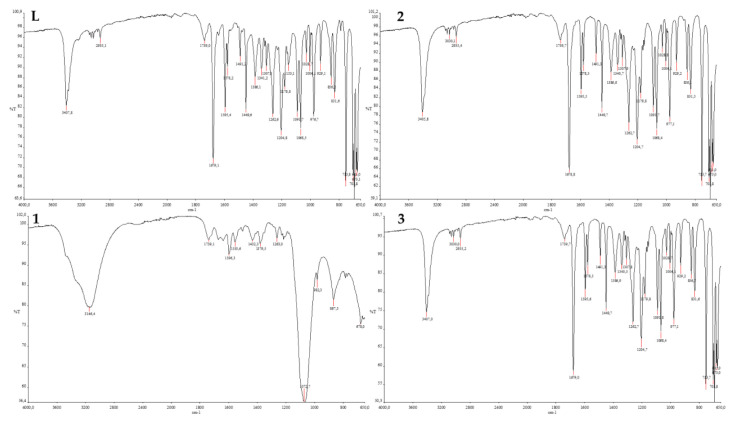
FTIR spectrum of L, 1, 2, and 3.

Infrared spectroscopy is highly effective for examining organic compounds, especially those with asymmetric structures and polar functional groups. Compound L had a –OH broad absorption peak at approximately 3200–3400 cm^−1^. NH stretching was detected at as a sharp peak at 3407 cm^−1^. The aromatic C–H stretching was observed above 3000 cm^−1^. The aliphatic C–H stretching was identified at 2935 cm^−1^. The imine (C=N–) group was detected at 1679 cm^−1^. The characteristic pyrimidine peak was observed at 1595 cm^−1^ and O=S=O signals were observed at 1307 and 1153 cm^−1^ (Liu et al., 2021). Interestingly, when the FTIR spectra of L and substances 1, 2, 3, and 4 were compared, it was observed that there was no change in the fundamental peaks belonging to L for 2, 3, or 4. The peak at 1153, which is exclusive to L, was not observed in the metal complexes. The absence of the signal at 1153, belonging to the –SO_2_ group, in the metal complexes may indicate that the metal interacts with the –SO_2_ group. However, for compound 1 a peak of imine was not detected and a broad –OH signal was observed (Figure 6). FTIR analyses are not only a crucial analytical method for the characterization of metal complexes; they also provide valuable information regarding the stability of structures. The analyses conducted indicated that the structures could remain stable.

### 3.3. Density functional theory (DFT) calculations

The geometric optimization of a promising molecule was carried out using DFT with Gaussian 09, specifically at the B3LYP/6-31+G level of theory (Bauschlicher and Partridge, 1995; Bilge et al., 2009). The energies of the lowest unoccupied molecular orbital (LUMO) and the highest occupied molecular orbital (HOMO) were investigated. Analysis of the DFT results indicated the chemical reactivity sequence of compound L. Geometry optimization, aimed at determining the molecule’s stability and its lowest energy configuration, is a fundamental initial procedure in quantum chemical studies. These calculations were performed using DFT methods (B3LYP) combined with a 6-31+G basis set within the software Gaussian 09W (Frisch et al., 2016). Table 1 displays the HOMO and LUMO energies for molecule L, as obtained from the DFT calculations at the B3LYP/6-31+G level. By calculating these energies, the relative stability and reactivity of the molecule were determined. A smaller HOMO–LUMO gap indicates a molecule that is less stable and has more reactivity. The energy gap of L implies that this molecule has potential reactivity.

**Figure 7:**
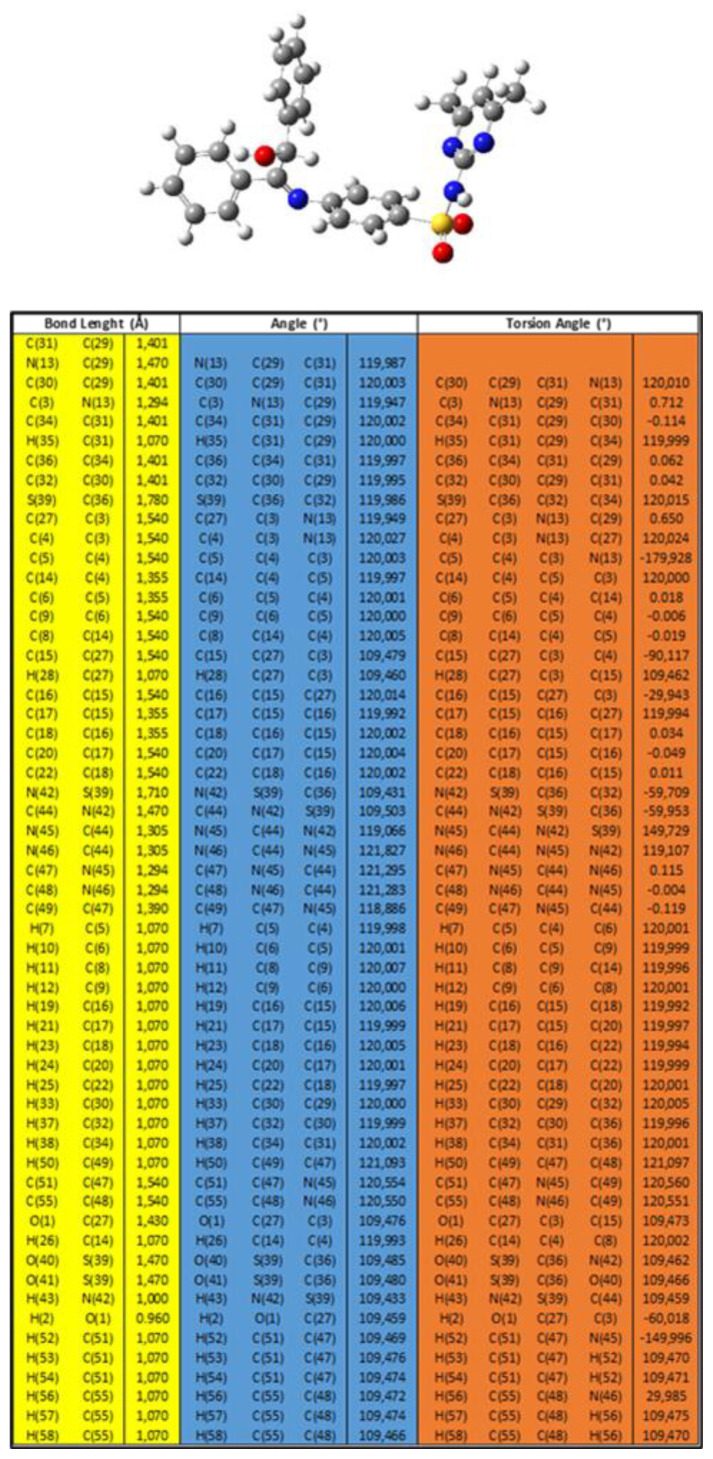
Bond length, angle, and torsion angle of L.

Detailed geometric data, such as bond lengths and angles for the optimized molecule L, are presented in Figure 7. Frontier molecular orbitals were analyzed to assess the electrical properties and electron transport potential of the compounds. These orbitals significantly impact the molecular characteristics and biological activities of the substances. The energies of the LUMO and HOMO are indicative of the electron-accepting and electron-donating capabilities, respectively. Based on Figure 6, the calculated imine (C=N) bond length was found to be close to the required value.

**Figure 8:**
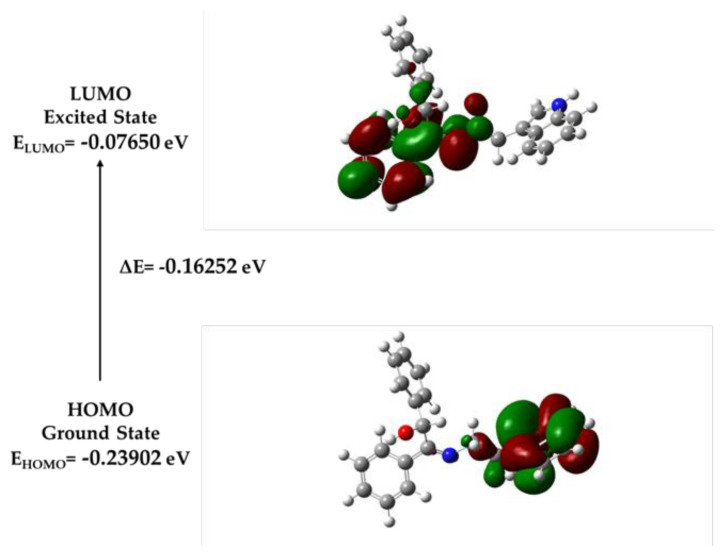
HOMO and LUMO energies of L.

The energies of the LUMO and HOMO are essential in determining a molecule’s chemical reactivity and stability. A smaller band energy gap (ΔE) between these orbitals indicates higher reactivity towards other substances. This is because a reduced energy gap facilitates charge transfer processes during chemical reactions (Govindarajan and Karabacak, 2012; Mumit et al., 2020). Compound L, which has a small band energy gap, demonstrates high reactivity. In this compound, the HOMO is located on the sulfamethazine side, while the LUMO is on the benzoin structure. The delocalized electrons within the HOMO suggest that electron movement is easier within the molecule, leading to better intramolecular charge transfer (Figure 8).

### 3.4. Antibacterial activity

In the present study, all synthesized compounds (L, 1, 2, 3, 4) were evaluated for their antibacterial and antifungal properties. Their minimum inhibitory concentrations (MICs) in μg/mL were determined against *E. coli* ATCC 8739, *S. aureus* ATCC 6538, *P. aeruginosa* ATCC 9027, and *C. albicans* ATCC 10231. Ciprofloxacin was used as a reference drug. For *E. coli*, Cu^2+^ containing Schiff base-metal complexes 1 (1031.25 μg/mL) exhibited a better MIC value than L (1250.25 μg/mL). The other compounds, 2 (>3250 μg/mL), 3 (2500 μg/mL), and 4 (1312.50 μg/mL), had higher MIC values than L (Table 2). The inhibitory potentials of the compounds for *S. aureus* were also investigated. In similar manner, compound 1 (1031.25 μg/mL) had a lower MIC value than L (>1250 μg/mL). The other compounds, 2 (>3250 μg/mL), 3 (2500 μg/mL), and 4 (2625 μg/mL), had higher MIC values than L (Table 2). These results indicate no consistent trend that metal complexes uniformly outperform L for *S. aureus*; in fact, only the copper-containing complex 1 demonstrated better inhibitory capacity. The last antibacterial activity test was conducted for *P. aeruginosa*. All compounds except 2 (>3250 μg/mL) had better MIC values than L (>1250 μg/mL). The best activity was observed for compound 3 (625 μg/mL). The MIC value of compound 4 (656.25 μg/mL) was very close to that of compound 3 (Table 2). Ciprofloxacin was used as reference drug for antibacterial activity; none of the compounds had lower MIC values than ciprofloxacin for the three antibacterial assays. Investigating the antifungal activity against *C. albicans* revealed that all compounds (1, 2, 3, 4) had better MIC activity than L (656.25 μg/mL).

The best activity was found for copper-based compound 1 (257.81 μg/mL). The MIC values of the zinc- and cobalt-based compounds 3 (312.50 μg/mL) and 4 (328.12 μg/mL) also showed similar promising activity. In summary, Schiff base-metal complexes generally exhibited enhanced antibacterial and antifungal activities compared to L.

Zinc-based compound 3 demonstrated notable antibacterial activity against *P. aeruginosa*, with a MIC value that was half of that observed for L, representing the best antibacterial result in our study. Additionally, the antifungal activity against *C. albicans* showed significant improvement, with copper-based compound 1 achieving a MIC value that was one-fifth that of L, marking the lowest MIC observed in the study. These findings underscore the potential of Schiff base-metal complexes in developing potent antimicrobial agents.

## 4. Conclusion

We successfully synthesized and characterized a novel Schiff base (compound L) and metal complexes of it with Cu^2+^, Co^2+^, Ni^2+^, and Zn^2+^. The synthesized compounds were evaluated for their antimicrobial properties, revealing that Schiff base-metal complexes generally exhibit enhanced antibacterial and antifungal activities compared to the ligand alone. Specifically, the copper-based complex 1 showed superior activity against *E. coli* and *C. albicans* with the lowest MIC values observed in the study, while zinc-based compound 3 demonstrated notable antibacterial activity against *P. aeruginosa*. These results underscore the importance of metal ions in enhancing the antimicrobial efficacy of Schiff base complexes and suggest that such compounds have significant potential in developing new antimicrobial agents. Further research should focus on the detailed mechanisms of action and exploring additional metal ions to optimize their bioactive properties.

## Figures and Tables

**Table 1:** Gaussian-based calculations of frontier molecular orbital energies of L.

HOMO (eV)	LUMO (eV)	Energy gap ΔE (eV)
−0.23902	−0.07650	0.16252

**Table 2:** Antibacterial and antibacterial activities (MIC values μg/mL) of compounds L, 1, 2, 3, and 4.

Compound	*L*	1	2	3	4	Ciprofloxacin
***E.coli***	1250	1031.25	>3250	2500	1312.50	0.38
***S. aureus***	>1250	1031.25	>3250	2500	2625	12.20
***P. aeruginosa***	>1250	1031.25	>3250	625	656.25	3.05
***C. albicans***	>1250	257.81	812.50	312.50	328.12	-
